# Physical aggression victimization among Brazilian adolescents: Associated factors and the role of school policies based on 2015 PeNSE data

**DOI:** 10.1590/1984-0462/2026/44/2025237

**Published:** 2026-05-11

**Authors:** Rinelly Pazinato Dutra, Yasmin Marques Castro, Andressa Munhoz Sá, Cleonice Santos do Amaral Bilharva, Michael Pereira da Silva

**Affiliations:** a Universidade Federal do Rio Grande, Rio Grande, RS, Brazil.

**Keywords:** Aggression, Policy, Adolescents, Students, Schools, Agressão, Política, Adolescente, Estudantes, Escolas

## Abstract

**Objective:**

The aim of this study was to analyze the prevalence, associated factors, and the relationship between the existence of school violence prevention policies and physical aggression victimization among Brazilian adolescents.

**Methods:**

Cross-sectional study based on data from the 2015 National School Health Survey (PeNSE). The sample included 15,954 adolescents aged 11–19 years, enrolled in public and private schools across all regions of Brazil. Victimization was measured through self-reported experiences of physical aggression in the past 12 months. Sociodemographic, behavioral, relational, and schoolrelated variables were analyzed. The association between these variables and victimization was estimated using Poisson regression with robust variance.

**Results:**

The prevalence of physical aggression victimization was 17.1%. Factors associated with higher prevalence included male sex, Black or other non-White ethnicities, reports of bullying, alcohol and cigarette use, high screen time, and being physically active. In contrast, older age and having a larger number of friends were protective factors. The presence of integrated actions with primary health care was associated with a lower likelihood of victimization (PR 0.82; 95%CI 0.74–0.90; p<0.001), whereas formal school policies showed no significant association.

**Conclusions:**

Physical aggression victimization among adolescents is a relevant and multifactorial problem. Intersectoral actions between health and education have the potential to reduce school violence, whereas isolated policies are ineffective without community engagement and a contextualized approach.

## RESUMO

**Objetivo:**

Analisar a prevalência, os fatores associados e a relação entre a existência de políticas escolares de prevenção à violência e a vitimização por agressão física entre adolescentes brasileiros.

**Métodos:**

Estudo transversal baseado em dados da Pesquisa Nacional de Saúde do Escolar (PeNSE) de 2015. A amostra incluiu 15.954 adolescentes de 11 a 19 anos, matriculados em escolas públicas e privadas de todas as regiões do Brasil. A vitimização foi mensurada por autorrelato de agressões físicas nos últimos 12 meses. Foram analisadas variáveis sociodemográficas, comportamentais, relacionais e escolares. A associação entre essas variáveis e a vitimização foi estimada por regressão de Poisson com variância robusta.

**Resultados:**

A prevalência de vitimização por agressão física foi de 17,1%. Os fatores associados a maior prevalência incluíram: sexo masculino, raça/cor da pele preta ou outras etnias, relato de bullying, uso de álcool e cigarro, tempo de tela elevado e ser fisicamente ativo. Por outro lado, idade mais avançada e maior número de amigos foram fatores protetores. A presença de ações integradas com a Atenção Primária à Saúde foi associada a menor probabilidade de vitimização (RP=0,82; IC95% 0,74–0,90; p<0,001), enquanto políticas escolares formais não mostraram associação significativa.

**Conclusões:**

A vitimização por agressão física entre adolescentes é um problema relevante e multifatorial. Ações intersetoriais entre saúde e educação demonstram potencial para reduzir a violência escolar, enquanto políticas isoladas carecem de efetividade sem engajamento comunitário e abordagem contextualizada.

## INTRODUCTION

 Violence is a multifaceted problem encompassing various forms of intentional power or force, which may result in physical harm, emotional trauma, or disruptions to individual development.^1,2^ During adolescence, a stage marked by intense biopsychosocial changes, exposure to violence, whether direct or indirect, can generate long-lasting adverse effects, affecting emotional well-being, academic performance, and social relationships, as well as increasing the risk of involvement in violent behaviors.^2-5^

 National and international data reveal high-prevalence rates of violence among adolescents, both as victims and perpetrators. In Brazil, the National School Health Survey (PeNSE) identified an increase in aggressive behavior among adolescents, from 12.9% in 2009 to 18.2% in 2019.^6,7^ According to the 2014 Violence and Accident Surveillance Survey (VIVA), 815 adolescents received urgent or emergency care in Brazilian capitals due to aggression-related victimization.^8^ In the United States, the 2013–2023 Youth Risk Behavior Survey (YRBSS) Trend Report showed that threats or injuries with weapons at school rose from 6 to 9%, and absences due to safety concerns increased from 7 to 13%. Bullying cases grew from 15% in 2021 to 19% in 2023.^9^ In Europe, similarly, concerning prevalence rates have been reported, such as in Finland, where 13.2% of students were frequent victims of peer aggression, and in Norway, where one in six adolescents was involved in physical fights.^10,11^

 Several factors increased vulnerability to physical aggression victimization among adolescents. Characteristics such as depressive symptoms and a previous history of aggressive behavior significantly increase the risk.^4,10^ The socio-spatial context is also important: adolescents aged 15–19 are more frequently victimized in public spaces by unknown aggressors, while younger adolescents (10–14 years) are predominantly victimized at school, often by peers.^8,10^ Behavioral patterns, such as alcohol/tobacco consumption, attention problems, and poor academic performance, coexist with an aggressor–victim cycle, in which aggressive behaviors appear to predict subsequent victimization.^4,11^ Gender differences are also evident: while boys are more often involved in physical fights, girls report greater victimization by peers and a higher occurrence of aggression at home.^8,10,11^ These findings suggest heightened vulnerability among adolescents in violent urban contexts, particularly those with histories of aggressiveness and chronic exposure.^4,8,12^

 Preventing school violence requires integrated approaches combining protective factors at multiple levels. Interventions fostering positive student–teacher relationships can reduce peer aggression, improve psychological well-being, and enhance academic performance.^13^ Globally, strategies for addressing violence involve initiatives by schools, civil society organizations, and public policies.^14-16^ In Brazil, the School Health Program (PSE) exemplifies this intersectoral approach. Developed through a partnership between the Ministries of Health and Education, the PSE aims to promote comprehensive health and prevent harm among public school students, with violence prevention as a strategic axis.^17^ Through collaboration between schools and primary health care (PHC) teams, the program seeks to strengthen a culture of peace in educational settings.^17-19^

 Given the above, violence and aggression are prevalent, multifactorial, and complex phenomena, with harmful consequences across multiple aspects of adolescents’ lives. School-based research is essential to investigate adolescent health conditions. By addressing issues related to violence, PeNSE editions can substantially contribute to monitoring the situations experienced by Brazilian students. This study aims to analyze the prevalence, associated factors, and the relationship between the existence of school violence prevention policies and physical aggression victimization among Brazilian adolescents. 

## METHOD

 A cross-sectional study was conducted using secondary data from the 2015 PeNSE.^20^ PeNSE 2015 used a complex, stratified, and clustered sampling plan with two independent samples. Sample 1, composed of 9th-grade students, was stratified into 53 geographic domains (the 26 state capitals and the Federal District; the other municipalities in each state; the five major regions; and Brazil), covering 675 municipalities and 3160 schools, resulting in 102,072 valid questionnaires. Sample 2, consisting of adolescents aged 13 to 17, was stratified by the five major regions of the country, including 179 municipalities and 380 schools, with 10,926 valid questionnaires. In both samples, sample weights were applied to correct for different selection probabilities, ensuring the national and regional representativeness of Brazilian adolescents. For the present study, we considered students aged 11 to 19 years from the PeNSE 2015 dataset. Of the 19,402 students in this age group enrolled in 371 public and private schools across all Brazilian geographic regions, PeNSE collected data from 16,608 adolescents, resulting in a response rate of 85.6%. Due to errors or incomplete data, 654 participants were excluded from the present study, totaling a final sample of 15,954 adolescents. 

 The outcome analyzed was victimization by physical aggression, which was assessed through the question: "In the past 12 months, how many times have you been physically assaulted?" Adolescents could respond from "None" to "12 or more times." The responses were categorized dichotomously (yes or no), considering as positive cases those adolescents who reported having been assaulted at least once during the period, and as negative those who reported no occurrence.^20^

 Independent variables included sociodemographic characteristics (sex, age, and race/skin color), behavioral aspects (use of alcohol, cigarettes, and illicit drugs in the past 30 days, physical activity, and daily screen time), relational aspects (number of friends and bullying report), and school characteristics (type of school administration and presence of institutional policies).^20^

 The variable sex was assessed dichotomously (male or female). The variable age was presented in descriptive analyses by age groups (11–12, 13–14, 15–16, and ≥17 years), but in regressions, it was kept as a continuous variable in complete years. The variable race/skin color was self-reported according to IBGE categories and, for analytical purposes, grouped (white, Black, brown, others).^20^

 The number of friends was self-reported and classified as "none," "1 or 2," and "3 or more." The question about bullying, "Have you ever been bullied?", was measured with a dichotomous response (yes/no). The use of psychoactive substances was assessed based on questions about consumption in the past 30 days. The variables alcohol, cigarette, and illicit drug consumption were operationalized dichotomously (yes or no), considering as positive the indication of at least one day of use in the period.^20^

 In the block of questions about physical activity, the questions addressed the amount, location, and type of physical activity, and adolescents who accumulated at least 300 minutes per week were considered active, according to current recommendations. Screen time was estimated through the daily time spent watching television on weekdays, using the question: "On a typical weekday, how many hours a day do you watch TV? (do not consider Saturdays, Sundays, and holidays)," being considered high when equal to or greater than 2 hours per day.^20^

 PeNSE also collected data from participating schools through a targeted questionnaire.^20^ The type of school was assessed as public or private administration. In this study, the presence of school policies was analyzed through four variables:

- Policies against bullying: Assessed through the question: "Does the school have any policy, regulation, or written rule that prohibits bullying on its premises?"
- Policies against fights: Assessed through the question: "Does the school have any policy, regulation, or written rule that prohibits fights on its premises?"
- Policies against physical punishment of students by school staff: Assessed through the question: "Does the school have any policy, regulation, or rule that prohibits physical punishment of students by teachers or staff on its premises?"
- Integrated actions with PHC: Assessed through the question: "Does the school carry out joint actions with the Basic Health Unit, Family Health Team, or Primary Health Care Team?"

Analyses were performed using Stata MP 14.1 software, considering the complex sampling design with application of sample weights and cluster (schools) error control. Initially, descriptive analysis of the variables was carried out using absolute and relative frequencies. Subsequently, Poisson regression with robust variance was applied to estimate prevalence ratios (PR) and respective 95% confidence intervals (95%CI), to assess the association between independent variables and victimization by physical aggression. Crude and adjusted models were performed, with the final model including only variables that showed statistical significance (p<0.05) in the bivariate analyses. The choice of Poisson regression with robust variance is justified as an appropriate alternative to estimate PR in cross-sectional studies with dichotomous outcomes. 

 The 2015 PeNSE was approved by the National Research Ethics Committee under No. CAAE: 38990714.6.0000.0008. All participants provided informed consent through signed forms from their legal guardians, along with assent forms signed by the adolescents themselves. 

## RESULTS

Table 1 presents the characteristics of the study population. A total of 15,954 adolescents were analyzed, with a predominance of females (50.3%), age under 15 years (56.5%), and brown race/skin color (40.7%). Most attended public schools (74.2%) and reported having three or more friends (77.8%). Approximately 47% of adolescents reported having experienced bullying, with a slightly higher prevalence among females (48.5 vs. 45.3%; ꭓ^2^=15.95, p<0.001). 

**Table 1:** Sociodemographic characteristics, bullying, number of friends, school policies, and occurrence of physical aggression among Brazilian adolescents, 2015 National School Health Survey, Brazil (n=15,954).

		Overall sample		Male		Female		ꭓ^2^	p-value
		n	%	n	%	n	%		
Female sex		8021	50.3	-	-	-	-	-	-
Age group (years old)									
	11–12	4486	28.1	2121	26.7	2365	29.5	22.42	<0.001
	13–14	4528	28.4	2273	28.7	2255	28.1		
	15–16	4580	28.7	2284	28.8	2296	28.6		
	≥17	2360	14.8	1255	15.8	1105	13.8		
Race-skin color									
	White	6384	40.1	3307	41.7	3077	38.4	80.59	<0.001
	Black	1830	11.5	1036	13.1	794	9.9		
	Brown	6488	40.7	3002	37.9	3486	43.5		
	Others	1234	7.7	579	7.3	655	8.2		
School administration									
	Public	11,846	74.2	5926	74.7	5920	73.8	1.67	0.196
	Private	4108	25.8	2007	25.3	2101	26.2		
Number of friends (n=15,937)									
	None	600	3.8	356	4.5	244	3.0	75.73	<0.001
	1 or 2	2941	18.4	1274	16.1	1667	20.8		
	3 or more	12,396	77.8	6292	79.4	6104	76.2		
Being bullied		7479	46.9	3593	45.3	3886	48.5	15.95	<0.001
Alcohol use		3396	21.3	1704	21.5	1692	21.1	0.35	0.552
Cigarette use		768	4.8	420	5.3	348	4.3	7.95	0.005
Illicit drugs use		1383	8.7	753	9.5	630	7.8	15.51	<0.001
Physical activity ≥300 min/week		5192	32.5	3226	40.7	1966	24.5	474.15	<0.001
Screen time ≥2 hours/day		10,081	63.2	5027	63.4	5054	63.0	0.22	0.639
School policy against bullying		14,282	89.5	7132	89.9	7150	89.1	2.47	0.116
School policy against physical fights		15,120	94.8	7516	94.7	7604	94.8	0.03	0.870
School policy against physical punishments		14,132	89.7	7098	89.5	7214	89.9	0.93	0.334
Integrated actions with PHC		9923	62.2	4971	62.7	4952	61.7	1.45	0.229
Suffered aggression in the last 12 months		2735	17.1	1480	18.7	1255	15.7	25.43	<0.001

PHC: Primary Health Care. Source: The authors.

 Regarding substance use, 21.3% reported alcohol consumption in the past 30 days, 4.8% reported cigarette use, and 8.7% reported illicit drug use during the same period. Cigarette use (5.3 vs. 4.3%, ꭓ^2^=7.95, p=0.005) and illicit drug use (9.5 vs. 7.8%, ꭓ^2^=15.51, p<0.001) were higher among male adolescents. Only 32.6% of adolescents were classified as physically active (≥300 minutes per week), with a higher proportion among males (40.7 vs. 24.5%; ꭓ^2^=474.15, p<0.001). Additionally, 63.2% of adolescents reported high screen time (≥2 hours per day watching TV on weekdays). 

 Regarding school policies, 89.5% of adolescents attended schools that reported having formal anti-bullying policies, 94.8% studied in schools with rules against physical fights, 89.7% were in institutions that prohibited physical punishment by teachers or staff, and 62.2% attended schools that implemented integrated actions with PHC. Victimization by physical aggression in the past year was reported by 17.1% of adolescents, being more prevalent among males (18.7 vs. 15.7%; ꭓ^2^=25.43, p<0.001). 

Table 2 presents the regression models for factors associated with victimization by physical aggression among Brazilian adolescents. Only statistically significant variables (p<0.05) remained in the final model. Male adolescents had a higher probability of victimization by physical aggression (PR 1.20; 95%CI 1.08–1.32) compared with females. Regarding race/skin color, adolescents who self-identified as Black (PR 1.23; 95%CI 1.06–1.43) or belonging to other ethnicities (PR 1.21; 95%CI 1.03–1.41) had a higher probability of victimization compared with white adolescents. Reporting having been bullied was associated with more than twice the likelihood of victimization (PR 2.08; 95%CI 1.85–2.34). 

**Table 2:** Factors associated with physical aggression victimization in Brazilian adolescents, 2015 National School Health Survey, Brazil (n=15,954).

		Initial model		Final model	
		PR (95%CI)	p-value	PR (95%CI)	p-value
Male sex		1.20 (1.08–1.32)	<0.001	1.20 (1.08–1.32)	<0.001
Age groups*		0.87 (0.81–0.93)	<0.001	0.87 (0.81–0.93)	<0.001
Race-skin color					
	White	1		1	
	Black	1.24 (1.06–1.45)	0.006	1.23 (1.06–1.43)	0.007
	Brown	1.01 (0.90–1.14)	0.843	1.01 (0.90–1.13)	0.919
	Others	1.21 (1.03–1.41)	0.020	1.21 (1.03–1.41)	0.020
School administration					
	Private	1		-	
	Public	0.93 (0.83–1.05)	0.264	-	
Number of friends*		0.82 (0.76–0.89)	<0.001	0.82 (0.76–0.89)	<0.001
Being bullied		2.08 (1.85–2.34)	<0.001	2.08 (1.85–2.34)	<0.001
Alcohol use		1.39 (1.22–1.60)	<0.001	1.41 (1.24–1.62)	<0.001
Cigarette use		1.70 (1.39–2.08)	<0.001	1.76 (1.47–2.10)	<0.001
Illicit drugs use		1.09 (0.92–1.30)	0.312	-	
Physical activity ≥300 min/week		1.26 (1.14–1.40)	<0.001	1.26 (1.14–1.40)	<0.001
Screen time ≥2 hours/day		1.21 (1.08–1.35)	0.001	1.21 (1.08–1.36)	0.001
School policy against bullying		0.91 (0.76–1.10)	0.351	-	
School policy against physical fights		0.91 (0.70–1.19)	0.491	-	
School policy against physical punishments		1.15 (0.99–1.33)	0.069	-	
Integrated actions with PHC		0.83 (0.75–0.92)	<0.001	0.82 (0.74–0.90)	<0.001

* used as a numeric variable.

PHC: Primary Health Care. Source: The authors.

 Higher probability of victimization was also observed among adolescents who reported alcohol use (PR 1.41; 95%CI 1.241.62), cigarette use (PR 1.76; 95%CI 1.47–2.10), high screen time (PR 1.21; 95%CI 1.08–1.36), and those classified as physically active (PR 1.26; 95%CI 1.14–1.40). Conversely, older adolescents (PR 0.87; 95%CI 0.81–0.93) and those with a greater number of friends (PR 0.82; 95%CI 0.76–0.89) had a lower probability of victimization by physical aggression. 

 Concerning school policies, no statistically significant associations were observed between the presence of anti-bullying policies, rules against physical fights, or prohibitions on corporal punishment and victimization by physical aggression. However, adolescents attending schools with integrated PHC actions had an 18% lower probability of victimization (PR 0.82; 95%CI 0.74–0.90), a statistically significant result. 

## DISCUSSION

 This study analyzed the prevalence of victimization by physical aggression among Brazilian adolescents, its associated factors, and its relationship with the presence of school policies aimed at violence prevention, using data from the 2015 PeNSE survey. The results indicated that approximately 17% of adolescents reported being physically assaulted at least once in the past year. Factors associated with a higher probability of victimization included male sex, Black race/skin color, and other ethnicities, reporting being bullied, alcohol and cigarette use, high screen time, and being physically active. Conversely, older adolescents and those with more friends had a lower probability of victimization. Integrated actions with PHC were the only institutional factor associated with reduced aggression. In contrast, formal school policies against bullying, physical fights, and corporal punishment showed no significant association. 

 The prevalence of victimization by physical aggression identified in this study indicates that violence against adolescents remains an important public health problem in Brazil. This finding appears consistent with national and international estimates presented in the introduction, highlighting that exposure to physical aggression in this age group is a persistent phenomenon across school, family, and community settings.^6-11^ Considering this high-prevalence scenario, understanding factors that increase or reduce the risk of victimization is essential to guide policies and interventions. 

 Among these factors, male sex was associated with a higher probability of victimization by physical aggression. This result is consistent with part of the literature that identifies higher victimization among boys, although some studies report higher victimization among girls in specific contexts and forms of violence.^4,5,10,12^ Differences in measurement (e.g., peer victimization versus general physical aggression), sociocultural contexts, and study designs may explain this heterogeneity. Greater vulnerability of boys to victimization may be related to sociocultural constructions of gender, which normalize aggressive behaviors among male peers, particularly when they do not conform to expectations of masculinity, and in some contexts, minimize the severity of the violence they experience.^21,22^ Our results do not allow a deeper exploration of this gender inequality construct as an explanatory factor for differences between male and female adolescents; however, they point to the need for a better understanding of its impact on physical aggression among Brazilian male adolescents. 

 Adolescents identifying as Black or belonging to other racial/skin color groups reported higher frequencies of victimization by physical aggression, aligning with research highlighting the overlap between racial inequalities and exposure to violence.^23,24^ Evidence suggests that Black adolescents may experience more aggression due to a complex combination of factors, including interpersonal violence, racial discrimination, and structural racism, which create environments where these youth are disproportionately affected.^25,26^ This racialized vulnerability may reflect both greater exposure to violent contexts and discriminatory perceptions and less supportive school environments.^23,24^ These findings underscore the importance of public policies that consider intersections of race, territory, and schooling. 

 Behavioral and social factors were also associated with victimization. Reporting bullying was strongly linked to experiencing physical aggression, reflecting not only an overlap between different forms of violence but also a cycle of vulnerability, in which initial victimization increases susceptibility to subsequent assaults.^10,27^ Alcohol and cigarette use were likewise associated with higher prevalence of victimization, possibly indicating social contexts of vulnerability and exposure to risk. Literature suggests that adolescents who consume substances are more likely to be embedded in networks exposed to conflicts, increasing the likelihood of involvement in violent episodes, including as victims.^2,11^ These results are concerning, as adolescent alcohol and tobacco consumption is a public health problem. These risks are compounded when substance use contributes to victimization. 

 Findings related to physical activity require careful interpretation. Contrary to a meta-analysis identifying physical activity as a protective factor against bullying, physically active adolescents in this study exhibited a higher prevalence of victimization by physical aggression.^28^ Although counterintuitive, this may relate to exposure to competitive and hierarchical socialization spaces, such as school-based sports.^29^ Similarly, high screen time was associated with an increased risk of aggression, consistent with existing evidence.^28^ This pattern may reflect reduced qualitative social interaction, greater exposure to violent content, or diminished interpersonal skills, all of which can facilitate conflict situations.^27^ Future research should explore the contexts in which physical activity and screen time may act as potential risk factors, aiming to clarify the mechanisms that increase adolescents’ vulnerability to victimization by physical aggression. 

 Among protective factors, age stood out, as older adolescents had a lower probability of reporting victimization. Although this group may exhibit a greater propensity to perpetrate aggression toward younger peers, the protective effect of age pertains to vulnerability as a victim, potentially related to greater autonomy, socioemotional development, and coping strategies that reduce exposure to violence.^4,10-12^ Another protective factor was a higher number of friends, which may function as a social support network and reduce exposure. Evidence indicates that close, high-quality relationships provide emotional support and act as protective factors, whereas the absence or fragility of these connections increases vulnerability, particularly among adolescents with socioemotional difficulties.^10,27^

 The main finding of this study concerns the association between integrated actions with PHC and lower prevalence of victimization. This emphasizes the potential for coordination between health and education sectors, especially within the PSE, to enhance strategies that promote a culture of peace, care, and violence prevention in schools.^17,19^ Evaluations of the PSE in Brazilian schools have indicated its contribution to reducing fights and violent behaviors, reinforcing the importance of this intersectoral integration.^18^ The integration of PHC into daily school activities may facilitate attentive listening, formation of supportive relationships, and development of collective actions focused on mental health, mediation of social relationships, and conflict resolution. 

 On the other hand, the absence of association between formal school policies against bullying, physical fights, or corporal punishment and victimization suggests that isolated regulations do not guarantee effectiveness. Their implementation depends on the engagement of the school community, the training of professionals, and the capacity for conflict mediation. International evidence indicates that effective anti-violence policies require active involvement of educators, intersectoral approaches, and continuous monitoring of outcomes.^30^ In this regard, beyond normative or structural measures, prevention requires interventions that consider adolescents’ social context, including support for mental health and addressing risk behaviors.^2,14-16^

 This study has some limitations. As it is based on secondary and self-reported data, it is subject to recall and social desirability biases. Furthermore, PeNSE does not provide detailed information on the type, intensity, or location of the aggression experienced, which limits understanding of the dynamics of violence. It was also not possible to explore how integrated actions with PHC were operationalized in each school or to assess their intensity or frequency. Additionally, the cross-sectional design does not allow for causal inferences between the associated factors and victimization by physical aggression, restricting the interpretation of findings to associations. 

 Despite these limitations, the broad sample coverage and national representativeness provide robustness to the results, enabling identification of relevant patterns of victimization by physical aggression among Brazilian adolescents. This study identified that victimization by physical aggression was associated with sociodemographic and behavioral factors, including male sex, Black or other racial/ethnic backgrounds, substance use, bullying, high screen time, and regular physical activity. Conversely, school actions integrated with PHC were associated with lower victimization rates, highlighting the potential of synergistic work between schools and the public health system. These findings provide valuable evidence to guide the planning and improvement of public policies aimed at violence prevention. In this context, intersectoral policies, guided by a broad multidisciplinary approach, are essential to promote comprehensive care and strengthen violence prevention in school settings. 

## Data Availability

The database that originated the article is available with the corresponding author.
